# Modulating autophagy in mesenchymal stem cells effectively protects against hypoxia- or ischemia-induced injury

**DOI:** 10.1186/s13287-019-1225-x

**Published:** 2019-04-17

**Authors:** Chenxia Hu, Lingfei Zhao, Daxian Wu, Lanjuan Li

**Affiliations:** 1 Collaborative Innovation Center for Diagnosis and Treatment of Infectious Diseases, State Key Laboratory for Diagnosis and Treatment of Infectious Diseases, School of Medicine, First Affiliated Hospital, Zhejiang University, Hangzhou Zhejiang, People’s Republic of China; 2 Kidney Disease Center, First Affiliated Hospital, School of Medicine, Zhejiang University, Hangzhou Zhejiang, People’s Republic of China; 3 Key Laboratory of Kidney Disease Prevention and Control Technology, Hangzhou Zhejiang, People’s Republic of China; 4 Institute of Nephrology, Zhejiang University, Hangzhou Zhejiang, People’s Republic of China

## Abstract

In mammals, a basal level of autophagy, a self-eating cellular process, degrades cytosolic proteins and subcellular organelles in lysosomes to provide energy, recycles the cytoplasmic components, and regenerates cellular building blocks; thus, autophagy maintains cellular and tissue homeostasis in all eukaryotic cells. In general, adaptive autophagy increases when cells confront stressful conditions to improve the survival rate of the cells, while destructive autophagy is activated when the cellular stress is not manageable and elicits the regenerative capacity. Hypoxia-reoxygenation (H/R) injury and ischemia-reperfusion (I/R) injury initiate excessive autophagy and endoplasmic reticulum (ER) stress and consequently induce a string of damage in mammalian tissues or organs. Mesenchymal stem cell (MSC)-based therapy has yielded promising results in repairing H/R- or I/R-induced injury in various tissues. However, MSC transplantation in vivo must overcome the barriers including the low survival rate of transplanted stem cells, limited targeting capacity, and low grafting potency; therefore, much effort is needed to increase the survival and activity of MSCs in vivo. Modulating autophagy regulates the stemness and the anti-oxidative stress, anti-apoptosis, and pro-survival capacity of MSCs and can be applied to MSC-based therapy for repairing H/R- or I/R-induced cellular or tissue injury.

## Introduction

**Fig. 1:**
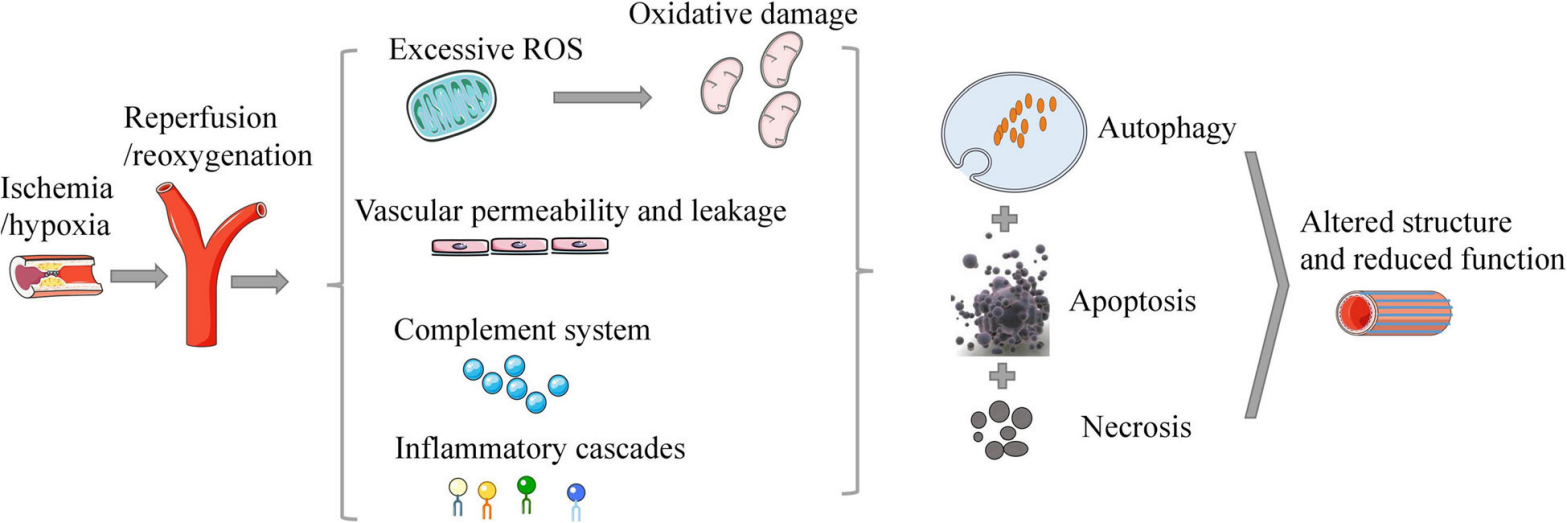
The potential mechanisms of I/R- and H/R-induced injury in organs

Although timely restoration of blood flow or oxygen has long been appreciated to reduce the injury in ischemic tissue by replenishment of cellular ATP, hypoxia-reoxygenation (H/R) and ischemia-reperfusion (I/R) injury, which aggravate the progression of organ dysfunction by reintroducing oxygen-rich blood to the impaired tissue [1], initiate autophagy and endoplasmic reticulum (ER) stress in mammals; consequently, they induce a string of damage in tissues or organs [2, 3]. During the ischemic period, ischemia or hypoxia is able to break down the endothelial cell barrier and increase vascular permeability and leakage by downregulating acti-adenylate cyclase activity and intracellular cAMP levels [4, 5]. The mitochondria then produce less ATP, leading to anaerobic metabolism, impairment of sodium-potassium pumps, and detachment of ribosomes [6]. In the ischemic stage, reactive oxygen species (ROS) can be produced from the xanthine oxidase system, NADPH oxidase system, mitochondrial electron transport chain, and uncoupled nitric oxide synthases and lead to cytokine cascades and pathological conditions [7, 8]. Reperfusion then upregulates the release of mitochondria-derived ROS [9], consequently disrupting normal ATP generation [10] and upregulating the mitochondrial permeability transition level [11, 12]. Excessive ROS cause oxidative stress, which promotes endothelial dysfunction, DNA damage, and local inflammatory responses [13]. Reperfusion predominantly triggers the release of intracellular damage-associated molecular pattern molecules (DAMPs) including high-mobility group box 1 and ATP and extracellular DAMPs including adenosine, thus promoting the accumulation of inflammatory cells including monocytes, dendritic cells, and granulocytes and activating the complement system [14–16]. Moreover, I/R also activates innate and adaptive immune responses including pattern-recognition receptors such as Toll-like receptors (TLRs) and inflammatory cell trafficking into the diseased organ (innate and adaptive immune activation) [17]. Moderate I/R simultaneously activate the recovery system after activation of autophagy, although autophagy does not clear all dysfunctional mitochondria during the intracellular perfusion stage [18]. If damage is severe, cell death may be induced via apoptotic or necrotic pathways, while a shorter duration of I/R may activate cell survival programs to control ROS generation and cell damage [19]. In addition, long-term I/R injury induces the generation of fibrotic scar tissue along with altered structure and reduced function in the injured site [20]. The current mechanisms of I/R- and H/R-induced injury should be further clarified for injury prevention throughout the entire process (Fig. 1).

Recently, the transplantation of mesenchymal stem cells (MSCs) has emerged as an effective strategy in regenerative medicine to repair injured organ function via regulating autophagy [21]. MSCs are multipotent cells that retain plasticity under standard culture conditions; these cells express high levels of makers CD105, CD73, and CD90 but rarely express CD45, CD34, CD14 or CD11b, CD79a or CD19, and HLA class II [22]. MSCs with high stemness and multipotency exhibit a high level of constitutive autophagy, while autophagy inhibitors prohibit proteolytic degradation and reduce the survival and differentiation of MSCs [23]. On the other hand, MSCs can undergo angiogenesis and secrete angiogenic factors in ischemic sites, thus promoting the recovery of I/R-injured tissues [24]. In addition, the immunosuppressive and pro-angiogenic effects of MSCs serve as important mechanisms for repairing tissue functions. MSCs not only inhibit macrophage-mediated inflammation and monocyte differentiation into immature dendritic cells [25] but also suppress the proliferation of B cells, antigen-primed T cells, and inactive natural killer cells [25–28]. Intriguingly, upregulating autophagy remarkably enhances the secretion of transforming growth factor (TGF)-β1 from MSCs and suppresses the proliferation of CD4^+^ T lymphocytes [29], whereas inhibiting autophagy reduces the responsiveness of T cells to mitogen interleukin (IL)-2 and increases the production of immunosuppressive prostaglandin E2 (PGE2) [30]. With such fascinating properties, MSCs may potentially be used for clinical applications in vessel repair and ischemic diseases and may be able to successfully treat ischemic tissues.

**Fig. 2:**
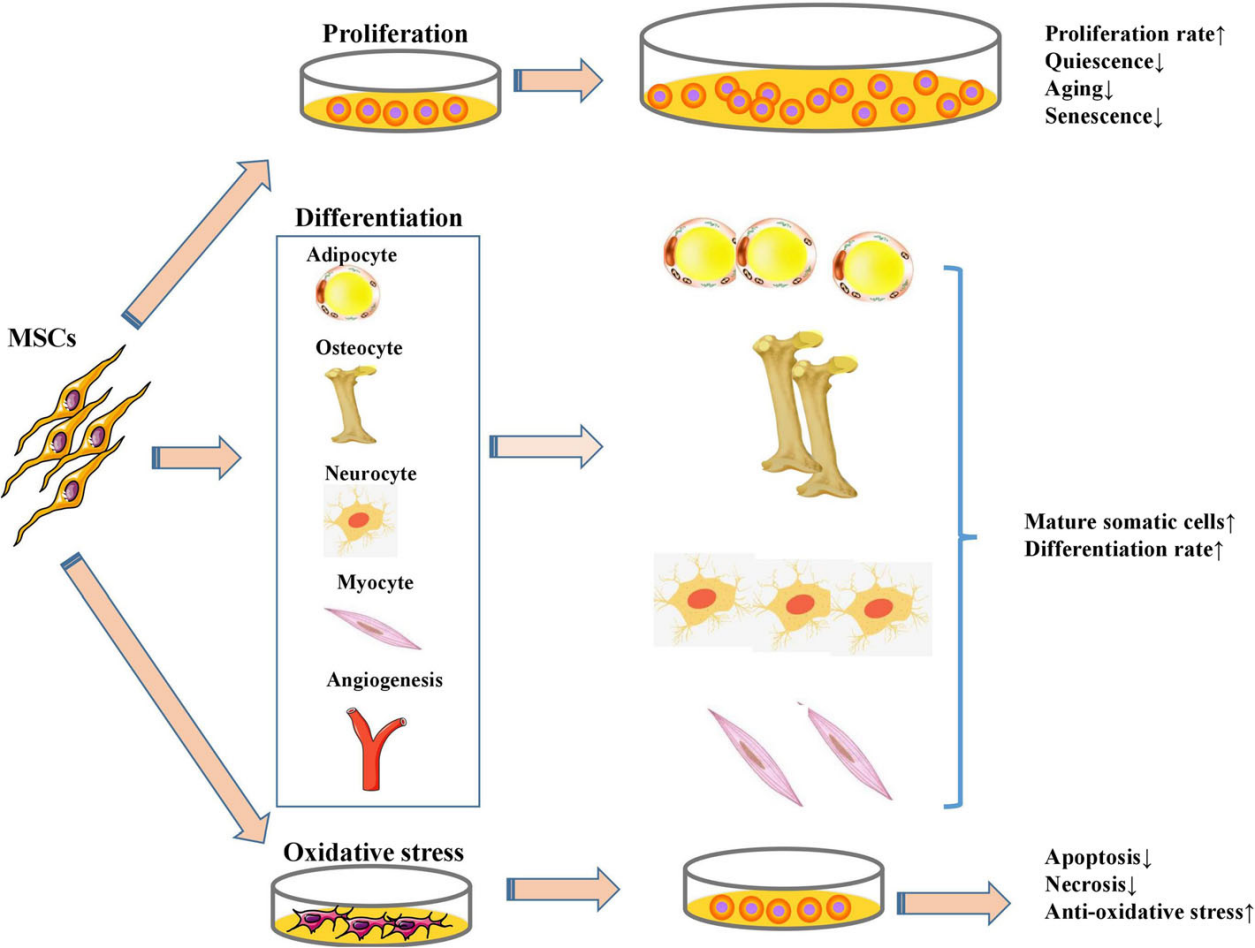
Modulating autophagy regulates the stemness, differentiation, survival, and apoptosis of MSCs

Autophagy is a type II programmed cell death [31] that can be activated to promote cell survival [32] or result in cell death [33] by stimulating various physiological and pathological factors. A basal level of autophagy occurs as a self-eating cellular process to degrade cytosolic proteins and subcellular organelles in lysosomes, recycle the cytoplasmic components, and regenerate cellular building blocks and energy, thus maintaining cellular and tissue homeostasis in all eukaryotic cells [34, 35]. Modulating autophagy regulates the stemness, differentiation, survival, and apoptosis of MSCs (Fig. 2). Although MSC transplantation has yielded promising results in treating H/R- or I/R-induced injury, the application of MSCs is limited by their low survival and low grafting potency. Th**e**refore, further efforts to increase the survival and activity of MSCs in vitro and in vivo will shed light on the treatment of H/R- or I/R-induced injury in multiple organs.

In this review, we have comprehensively summarized the modulation of autophagy for improving the therapeutic effects of MSC transplantation for repairing H/R- and I/R-induced injury. Further studies of strategies to control autophagic flux will augment the application of MSCs in tissue engineering and regenerative medicine.

## Autophagy process

**Fig. 3:**
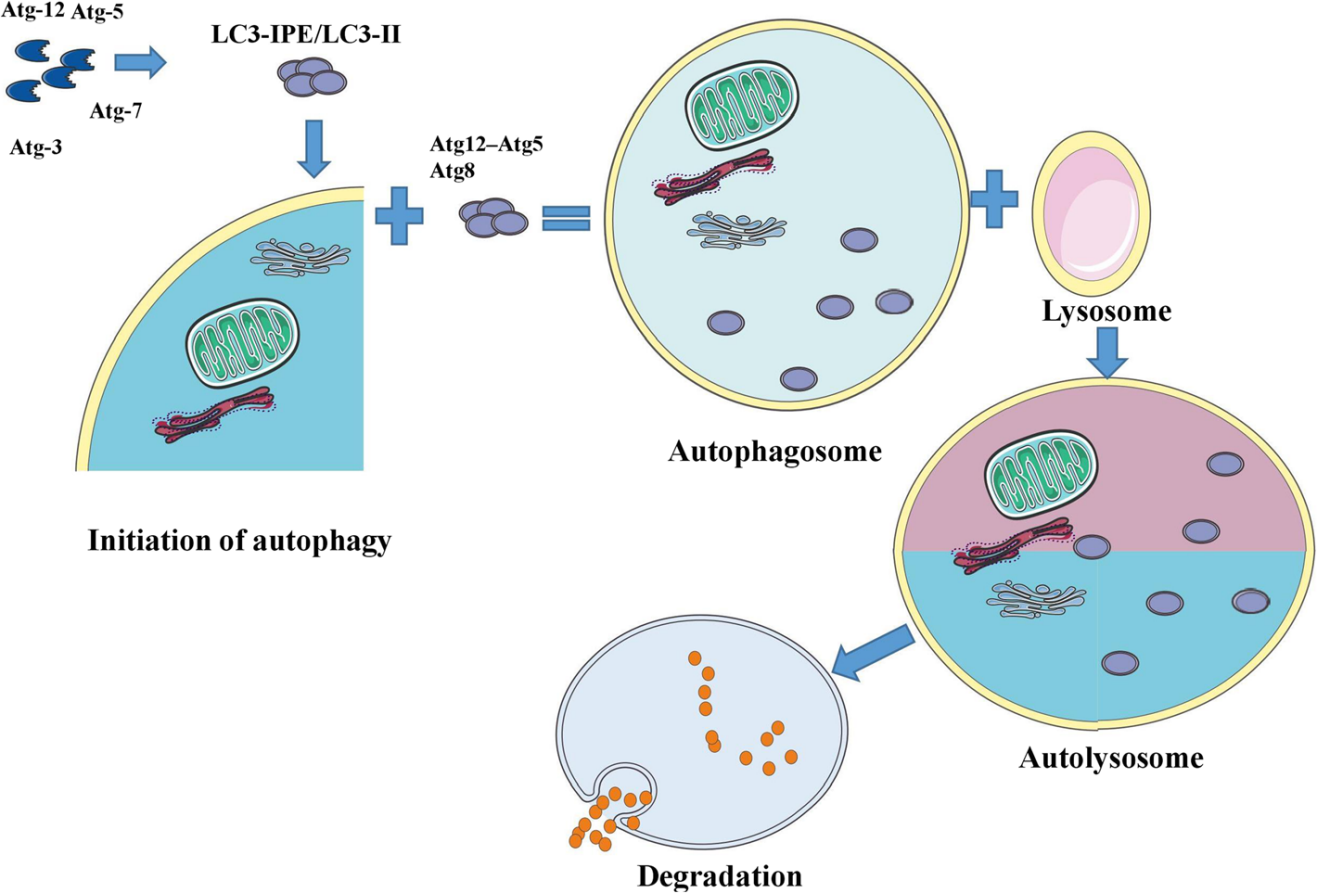
Macroautophagy serves as a quality control mechanism for proteins and organelles in mammals

In mammals, three types of autophagy, including macroautophagy [36], microautophagy [37], and chaperone-mediated autophagy (CMA) [38], serve as a quality control mechanism for proteins and organelles [39, 40]. Multiple signaling pathways regulate autophagy; the mammalian target of rapamycin (mTOR) and AMP-activated protein kinase (AMPK) pathways are the two major pathways that regulate autophagy in mammals [41]. In addition, other pathways, such as the AKT/PKB, p52, and inositol pathways, also play critical roles in modulating autophagy [42–44]. Macroautophagy can be divided into five steps, including initiation, vesicle nucleation, vesicle elongation, fusion, and degradation [45]. mTOR complex 1 (mTORC1), which regulates the phosphorylation and inactivation of UNC51-like kinase (ULK) 1/2 and Atg-13, downregulates the assembly of the ULK protein complex, which consists of Ulk1, Atg-13, FIP200, and Atg-101. Stressful conditions induce the dissociation of mTORC1 from the ULK protein complex, thus initiating macroautophagy [46]. Then, the Beclin-1/class III phosphatidylinositol-3-kinase (PI3K) complex forms, which are coordinated by the interactions of multiple proteins such as Beclin-1, Atg14, p150, Ambra1, endophilin B1, vacuolar protein-sorting 34, B cell leukemia/lymphoma 2, and the UV irradiation resistance-associated tumor suppressor gene; this complex helps activate PI3K to produce phosphatidylinositol-3-phosphate for vesicle nucleation [47]. Several Atg proteins (Atg5, Atg8, Atg12, and Atg16) are assembled into two ubiquitin-like markers and are tethered to the membrane of the pre-autophagosomes to elongate the vesicle. At the final stage, the vesicle closes to generate a complete autophagosome that fuses with an endosome or lysosome to generate an autophagolysosome that recycles materials or organelles [48]. However, a feedback mechanism terminates macroautophagy, as reactivation of mTOR generates proto-lysosomal vesicles that extrude from the autophagolysosome and develop into mature lysosomes [49]. This feedback inhibits excessive activation of autophagy under stressful conditions, thereby providing the full complement of the autophagy machinery. Microautophagy is a process through which cytoplasmic contents enter the lysosome through an invagination or deformation of the lysosomal membrane; this process helps maintain the normal size of organelles and membrane homeostasis and enhances cellular survival under stressful conditions [50]. Finally, CMA is a uniquely selective form of autophagy that degrades a wide range of substrate proteins after transporting them one by one into the lysosome [51]. Then, the no-longer-useful cytoplasmic, long-lived proteins and organelles, including mitochondria, peroxisomes, Golgi apparatus, and ER, are degraded by activated lysosomal enzymes. Impaired lysosomal activity leads to the accumulation of autophagosomes, thus impairing cellular function and activating caspase-mediated cell death [52]. In general, we refer to macroautophagy as autophagy in this review because it is the major type of autophagy in most live cells and tissues (Fig. 3).

### Modulation of autophagy to resist against oxidative stress

Slight autophagy is rapidly activated to recycle cytoplasmic materials to initiate a survival mechanism by producing energy under stressful conditions, including irradiation, pathogen infection, hypoxia, and starvation [53]; thus, this process certainly inhibits apoptosis and prolongs the survival time of engrafted MSCs in vivo. However, excessive activation of autophagy results in increased cell death, while inhibition of excessive autophagy allows MSCs to withstand the stressful conditions [54].

Autophagic flux is activated as a self-defensive mechanism at the early stage of hydrogen peroxide (H_2_O_2_)-induced injury in MSCs and is abolished after long-term exposure (i.e., 6 h) to a stressful condition, which results in the upregulation of caspase-3 and caspase-6 [55]. Pretreatment or cotreatment with rapamycin significantly ameliorates H_2_O_2_-induced cell death, while 3-MA exacerbates H_2_O_2_-induced cell apoptosis [55]. Treatment with H_2_O_2_ upregulates autophagy and apoptosis of MSCs in a concentration- and time-dependent manner, and augmenting the autophagy JNK inhibitor reduces apoptosis and increases the MSC survival rate in response to oxidative stress [56]. Since SDF-1β activation consequently activates the stromal cell-derived factor-1 (SDF-1)/CXC chemokine receptor 4 (CXCR4) signaling axis to improve MSC survival under oxidative stress, Herberg et al. demonstrated that the overexpression of SDF-1β protects MSCs from H_2_O_2_-induced cell death via upregulation of autophagy and downregulation of caspase-3-mediated apoptosis, even though SDF-1β overexpression did not exert any effect on the proliferation rate of MSCs in vitro [57]. Furthermore, MSCs cultured on chitosan substrates show spheroid formation and upregulated expression of the autophagosomal marker LC3 II in a Ca^2+^-dependent manner, and these changes are accompanied by a higher survival rate in response to H_2_O_2_ than in cells grown as a monolayer culture. In addition, chitosan substrates also increase the expression levels of the upper autophagy signal pathway-related proteins, including Ulk1, Atg-13, and autophagy/Beclin-1 regulator 1, both before and after the addition of H_2_O_2_ compared with general culture conditions [58]. Jun et al. argued that melatonin increases the levels of manganese superoxide dismutase (MnSOD) and catalase while suppressing H_2_O_2_-induced ER stress and autophagy to reduce the apoptosis rate of MSCs via upregulation of prion protein (PrP^C^) [59].

Irradiation induces ROS accumulation-associated DNA damage in MSCs, while rapamycin attenuates the damage via inducing autophagy [60]. Long-term incubation with palmitate impairs adaptive autophagic flux and enhances the accumulation of p62 in MSCs, while rapamycin-pretreated MSCs resist apoptosis via the ROS-JNK/p38 MAPK signaling pathway [61]. MSCs confront an inflammatory microenvironment after incubation with TNF-α/cycloheximide and show a decreased survival rate; rapamycin significantly improves cell survival, and knockdown of Beclin-1 and 3-MA accelerates the apoptotic progress of MSCs [62]. Tetramethylpyrazine (a traditional Chinese medicine) and eicosapentaenoic acid (an omega-3 long-chain polyunsaturated fatty acid) protect MSCs against dexamethasone-induced cytotoxicity, autophagy, and apoptosis in an AMPK/mTOR pathway-dependent manner [63, 64]. Moreover, application of vitamin C dramatically protects MSCs from Sin-1-induced oxidative stress via downregulation of autophagy, thus increasing the survival rate and proliferation of MSCs exposed to oxidative stress [65].

Gene modification of MSCs may enable the overexpression or knockdown of the key candidate genes of autophagy at high efficiency and specificity, thus improving the resistance of MSCs against oxidative stress. Carbon monoxide protects MSCs from oxidative stress-induced death via improving autophagy and mitophagy in normal MSCs; however, depletion of LC3B and Beclin-1 in MSCs increases oxidative stress-induced injury, decreases intracellular ATP, reduces mitochondrial membrane potential, and increases mitochondrial ROS, and this deficiency cannot be reversed by preconditioning with carbon monoxide [66]. Overexpression of superoxide dismutase 3 markedly reduces ROS production and increases the survival rate of MSCs via upregulation of autophagy under serum-deprived conditions. Moreover, superoxide dismutase 3 protects MSCs against serum deprivation-induced injury via modulation of AMPK/sirtuin 1, activation of extracellular signal-regulated kinase, and promotion of Forkhead box O3a trafficking to the nucleus [67]. Overexpression of carnitine palmitoyltransferase 1 C (CPT1C) increases MSC survival under glucose deprivation via enhancement of autophagy, which promotes the accumulation of lipid droplets and ATP production [68]. In contrast, knockdown of Beclin-1 increases the survival rate of MSCs in a TNF-alpha- and IFN-gamma-induced in vitro inflammatory environment via inhibition of autophagy and enhancement of Bcl-2 expression via the ROS/MAPK1/3 pathway [69].

## Hypoxia- and/or serum deprivation-induced injury

The in vitro oxygen concentration (approximately 20%) is much higher than that of the in vivo MSC niche; thus, engrafted MSCs promptly lose their activity and senesce to an apoptotic state after transplantation due to the stressful hypoxic environment [70]. However, hypoxia or serum deprivation may exert destructive or protective effects on MSCs according to the specific conditions (Table 1). An environment containing 0% O_2_ increases the autophagic flux via activation of the ERK1/2 pathway [71], and 1% O_2_ increases apoptosis via activation of the AMPK/mTOR pathway in MSCs; in contrast, 3-MA and the AMPK inhibitor compound C abolish hypoxia-induced apoptosis [72]. Ischemia induced by sodium azide (NaN_3_) and 2-deoxygluocose (2DG) blocks glycolysis and oxidative phosphorylation and consequently induces cellular autophagy and apoptosis in MSCs [73]. However, preconditioning with serum deprivation for 48 h reduces the energetic demand of MSCs and sends the cells into a quiescent state; then, the preconditioned MSCs maintain their viability and ATP levels via suppression of mTOR, activation of autophagy and enhancement of glycolysis even under exposure to 0.1% O_2_, and total glucose depletion for up to 14 days in vitro [74].

**Table 1:** Hypoxia or serum deprivation may exert destructive effects or protective effects on MSCs according to the specific conditions

Animal donor	MSC source	Treatment	Toxin	Autophagy	Mechanism	Effect	Reference
Mouse	Bone marrow	N/A	0% O_2_ (hypoxia or H/R)	↑	ERK1/2 pathway↑	Promote autophagic responses	[71]
Mouse	Bone marrow	N/A	1% O_2_ (H/SD)	↑	AMPK/mTOR signal pathway↑	Increase the apoptosis of MSCs	[72]
Rats	Bone marrow and adipose	N/A	NaN3 and 2DG	↑	VEGF↑; angiopoietin-1↓; extracellular matrix molecules↓	Enhance cell death	[73]
Human	Bone marrow	Serum deprivation	0.1% O_2_ and total glucose depletion	↑	mTOR↓; glycolysis↑	Maintain viability and ATP levels	[74]
Mouse	Bone marrow	1% O_2_	N/A	↑	Apelin/APJ/autophagy signaling pathway↑	Enhance MSC proliferation	[75]
Rat	Bone marrow	5% O_2_	Lipopolysaccharide	↑	HIF-1α↑	Improve cell activity and decrease apoptosis rate of MSCs	[76]
Mouse	Bone marrow	0.5% O_2_	H/SD	↑	AMP-activated protein kinase↑; mTOR↓	Protect MSCs from H/SD-induced injury	[77]
Human	Bone marrow	1%, 2%, 3%, 4% O_2_	N/A	↓	Mitochondrial activity↓	Decrease cellular size and increase cellular complexity	[78]
Rat	Bone marrow	ATV	H/SD	↑	AMPK/mTOR pathway↑	Enhance MSC survival	[79]
Rat	Bone marrow	Macrophage migration inhibitory factor (MIF)	H/SD	↑	AMPK/mTOR pathway↑; autophagy↑	Attenuate apoptosis of MSCs	[80]
Rat	Bone marrow	Overexpression of HIF-1α	OGD	↑	Autophagy↑; PI3K/AKT/mTOR pathway↓	Improve cell activity and reduce apoptosis rate of MSCs	[82]
Rat	Bone marrow	Sitagliptin	H/SD	↓	Bcl-2/Beclin-1 pathway↑	Attenuate apoptosis of MSCs	[83]

Transplantation of hypoxia-pretreated MSCs has become a primary method to improve the prognosis of various diseases via modulating autophagy. Preconditioning with 1% O_2_ enhances the proliferation of MSCs and increases the expression of hypoxia-inducible factor-1α (HIF-1α), apelin, Beclin-1, and LC3II/LC3I in a time-dependent manner [75]. Liu et al. argued that hypoxic pretreatment did not influence the cellular activities of MSCs under normal conditions but significantly improved cell activity and decreased the apoptosis rate of MSCs treated with lipopolysaccharide via enhancing HIF-1α-mediated autophagy [76]. Preconditioning with 0.5% O_2_ significantly upregulates autophagy to protect MSCs from hypoxia and serum deprivation (H/SD)-induced injury, while 3-MA and leptin-shRNA eliminate the protective effects via downregulating AMPK/mTOR-mediated autophagy [77]. Pezzi et al. argued that MSCs exposed to hypoxia reduce autophagy flux, which is accompanied by a smaller size, greater cellular complexity, and lower mitochondrial activity without altering oxidative stress in MSCs [78].

In addition to hypoxic pretreatment, some clinical drugs and growth factors can regulate autophagic flux in MSCs under hypoxic conditions. Atorvastatin (ATV) significantly activates autophagy via the AMPK/mTOR pathway to enhance MSC survival and reduce the apoptosis of MSCs under H/SD [79]. Pretreatment with macrophage migration inhibitory factor (MIF) protects MSCs against H/SD-induced apoptosis via upregulating autophagy and increasing the AMPK/mTOR pathway [80]. Because HIF-1α is a major regulator in mammals under hypoxic conditions, HIF-1α has been implicated as a major regulator of autophagy [81]. Overexpression of HIF-1α improves cell activity and reduces the apoptosis rate of MSCs, thus protecting MSCs from oxygen-glucose deprivation (OGD)-induced injury via activation of autophagy and inactivation of the PI3K/AKT/mTOR pathway [82]. However, sitagliptin (a dipeptidyl peptidase-4 inhibitor) effectively attenuates the apoptosis and autophagy of MSCs via regulating the Bcl-2/Beclin-1 pathway under an H/SD environment [83].

## I/R-induced injury

MSC-based therapy is widely used to treat I/R-induced injury to organs such as the heart, brain, liver, retina, and limb, but the transplanted MSCs often fail to engraft within the targeted tissue or organ, in part due to the poor survival of MSCs in response to hypoxia, nutrient starvation, inflammatory reactions, and oxidative stress. Autophagy may regulate MSC activities in vivo and repair I/R-induced injury in different tissues (Table 2).

**Table 2:** Autophagy may regulate MSC activities in vivo and repair I/R-induced injury in different tissues

Tissue	MSC source	Dose	Route	Animal	Treatment	Time point	Autophagy	Effect	Reference
Heart	Bone marrow	1 × 10^6^	Injection into the peri-infarcted areas	Mouse	1% O_2_	Transplantation immediately after MI	↑	Alleviate the apoptotic rate of cardiomyocytes; preserve heart function; eliminate fibrosis	[21]
	Bone marrow	Exosomes derived from 5 × 10^6^ cells	Injection into the anterior and lateral part of the visibly injured region	Rat	N/A	Transplantation prior to reperfusion	↑	Decrease the apoptosis rate of MSCs; reduce the myocardial infarct size	[91]
	N/A	2 × 10^5^	Injection into the peri-infarcted areas	Mouse	N/A	Transplantation at 30 min after MI	↓	Reduce infarct size; improve cardiac function	[92]
	N/A	5 μg MSC-derived exosomes	Injection into the peri-infarcted areas	Mouse	N/A	Transplantation at 30 min after MI	↓	Reduce infarct size; improve cardiac function	[92]
	Bone marrow	1 × 10^6^	Intramyocardial injection	Rat	Overexpression of Let-7b in MSCs	Transplantation immediately after MI	↓	Improve left ventricular function and microvessel density	[93]
Brain	Bone marrow	5 × 10^6^	Retro-orbital injection	Rat	N/A	Transplantation after 1 h and 24 h reperfusion	↓	Promote neurite growth and regeneration	[104]
	Bone marrow	2 × 10^6^	Intravenous injection	Rat	N/A	Transplantation at 30 min after TBI	↓	Promote histological and functional recovery in the brain	[105]
	Adipose	100 μg/kg/day of MSC-derived exosomes	Injection into the lateral cerebral ventricle	Rat	Overexpression of PEDF	Transplantation at 3 days prior to MCAO surgery	↑	Inhibit neuronal apoptosis; ameliorate cerebral I/R injury	[108]
	Bone marrow	1 × 10^6^	Injection into the striatum of the ipsilateral hemisphere	Rat	Overexpression of HIF-1α	Transplantation at 4 h after MCAO	↑	Reduce brain infarct volume; improve neurobehavioral outcome	[103]
	Bone marrow	5 × 10^6^	Injection into the superficial dorsal veins	Rat	Overexpression of HO-1	Immediate transplantation following the surgery	↑	Exert protective effects on liver grafts following reduced-size liver transplantation	[116]
	Bone marrow	1 × 10^6^	Portal vein	Rat	Overexpression of heat shock protein (HSP)	Transplantation at 60 min after reperfusion	↑	Decrease the levels of serum aminotransferases and Suzuki scores; improve cell survival and histopathology	[117]
Retina	Bone marrow	5 × 10^4^	Intravitreal transplantation	Rat	N/A	Transplantation at 24 h postischemia	↑	Eliminate ischemic damage in the retina	[120]
	Bone marrow	4 × 10^6^	Injection into the ischemic muscle along the femoral artery	Rat	5% O_2_	Transplantation immediately after surgery	↑	Upregulate the pro-angiogenic effects and therapeutic effects of engrafted MSCs in the lower limb of ischemic diabetic rats	[76]
	Adipose	1.0 × 10^7^	Injection into the left adductor muscle	Mouse	Apelin	Transplantation immediately after surgery and lasting for consecutive 14 days	↑	Enhance the survival of MSCs in ischemic hindlimbs; restore hindlimb blood perfusion; repair limb functions in peripheral arterial disease	[122]

### Acute myocardial infarction

AMI is a life-threatening disease induced by disrupted coronary blood flow that affects myocardial function; prolonged ischemia triggers molecular and structural changes in myocytes and leads to myocardial dysfunction. The incidence of AMI is 935 per 100,000 persons and remains the leading cause of mortality in the USA [84]. I/R-induced injury leads to heart failure in up to 40% of patients, and any delay in restoring coronary flow causes extensive cardiac cell death and results in ventricular remodeling in the injured heart tissue [85]. Although pharmacological agents or angioplasty is immediately used to reopen the occluded coronary artery after the onset of ischemic insult [86], the remaining inflammatory factors induced by AMI continue to impair myocytes in vivo. In vitro experiments have demonstrated that exposure to H/R upregulates autophagic flux in cultured rat neonatal cardiomyocytes [87] and H9C2 cells [88, 89]. Moreover, ischemia of differing severity leads to varied effects on autophagy modulation in cardiomyocytes under ischemic conditions. In cultured H9C2 cells, 2-deoxy-d-glucose induces mild ischemia and upregulates both autophagic flux and apoptosis, while sodium dithionate alone or with 2-deoxy-d-glucose induces apoptosis and necrosis without initiating autophagy [90].

Enhancing autophagy by hypoxia before MSC transplantation significantly improves the survival rate and the therapeutic effects of MSCs in repairing the ischemic myocardium, thus preserving heart function and eliminating fibrosis [21]. Injecting MSC-derived exosomes into rats that had undergone AMI markedly decreased the apoptosis rate of MSCs and reduced the myocardial infarct size via upregulating myocardial LC3B expression and activating the AMPK/mTOR and AKT/mTOR pathways [91]. However, Xiao et al. argued that the transplantation of MSCs and MSC-derived exosomes reduced the infarct size and improved the cardiac function via reducing apoptosis and autophagic flux in a mouse AMI model [92]. In addition, overexpressing Let-7b significantly decreased the apoptosis rate and increased the survival rate of MSCs by downregulating autophagy under high ROS conditions. Intramyocardial injection of these Let-7b-overexpressing MSCs significantly improved left ventricular function and microvessel density in AMI rats by directly targeting the caspase-3 signaling pathway [93]. The regulation of autophagy during I/R-induced myocardial injury appears to depend on the severity and duration of the ischemia. MSC transplantation regulates resident myocyte regeneration and heart function by regulating autophagy and ROS.

## Cerebral I/R injury

Even though the expression level of autophagosomes in the mature brain under normal conditions and starvation is always lower than in other organs, such as the heart, liver, pancreas, kidney, and skeletal muscle [94], modulating autophagy is vital for maintaining homeostasis and protein quality control in neurons. Autophagy enhances brain tissue damage at the early stage after focal brain ischemia but indispensably enhances the reconstruction of injured neurons and brain function by removing harmful protein aggregates and damaged organelles during the postischemic phase [95]. However, the authentic function of autophagy is still under debate, as autophagy plays different roles in ischemic brain injury according to brain maturity, the brain region, ischemia severity, ischemia stage and the timing of therapeutic interventions. In early studies, upregulated autophagy was widely accepted as a deteriorative factor for repairing brain function. Nitatori et al. first reported that cerebral ischemia increased the number of cathepsin B-immunopositive lysosomes in neurons via upregulating autophagy [96], and neonatal ischemia induced autophagy-mediated cell death in the absence of apoptotic markers during reperfusion [97, 98]. In contrast, human brain tissue from adult patients after traumatic brain injury (TBI) exhibits higher levels of autophagosomes and LC3-II than control tissue [99], and the levels of p62/sequestosome 1 and Beclin-1 are significantly higher in cerebrospinal fluid from children with severe TBI than in normal control cerebrospinal fluid [100]. Coculture with MSCs protected the cortical neurons from cell death via alleviating apoptosis-inducing factor-mediated parthanatos, attenuating receptor-interacting protein kinase (RIP) 1- and 3-mediated necroptosis, and downregulating caspase-3-mediated apoptosis, but coculture exerted no effects on the regulation of autophagy [101]. However, mild hypothermia enhanced autophagic flux and preserved the hippocampal neural function via downregulating lysosomal number and acidity, the ratio of autolysosomes to autophagosomes, and expression of LAMP2 in OGD-induced injury in hippocampal neurons [102]. Overexpression of HIF-1α protects MSCs from OGD-induced injury by enhancing cell viability, inhibiting apoptosis, and activating autophagy via activating the AMPK pathway and inhibiting the mTOR pathway [103].

To attenuate autophagy-induced cell death, Yin et al. transplanted MSCs via the retro-orbital route, which significantly promoted neurite growth and regeneration in rats with spinal cord I/R injury [104]. MSC transplantation also decreased the expression levels of LC3, Beclin-1, and connexin 43, thus decreasing autophagy and apoptosis-related neuronal cell death in the hippocampus after TBI [105]. Transplanting MSCs into a middle cerebral artery occlusion (MCAO) rat model significantly reduced the expression of the autophagy-associated proteins LC3 and Beclin-1 and consequently enhanced behavioral recovery, reduced the cerebral infarction volume, and downregulated the rate of apoptosis in rat neurons via activation of the PI3K/AKT/mTOR signaling pathway [106]. In contrast, other studies demonstrated that MSC transplantation protects the brain from I/R-induced injury by augmenting autophagy in vivo. MSC transplantation significantly increases the brain-derived neurotrophic factor level and reduces the activation of mTOR pathway; thus, MSC transplantation subsequently increases autophagy and exerts neuroprotection, attenuating the behavioral deficits after hypoxia-ischemia-induced injury in the brain [107]. Transplantation of HIF-1α-MSCs into MCAO rats significantly reduced brain infarct volume and improved neurobehavioral outcome via activating autophagy, inhibiting pro-inflammatory cytokine generation, and enhancing neurotrophin secretion [103]. In addition, transplantation of exosomes from pigment epithelium-derived factor (PEDF)-overexpressing MSCs at 3 days prior to MCAO surgery also ameliorated cerebral I/R injury by upregulating autophagy and inhibiting neuronal apoptosis [108].

### I/R-induced liver injury

Liver transplantation, partial hepatectomy, abdominal trauma, and hemorrhagic shock completely or partially interrupt liver blood flow and oxygen supply, followed by reperfusion to re-establish a new supply of blood and oxygen, but the reperfusion process aggravates the liver injury [109, 110]. Liver transplantation is the most effective treatment for patients with end-stage liver disease or patients with irreversible liver tumors [111]. However, long-term stimulation of excessive autophagy induced by severe ischemia in the liver destroys the structural organelles and leads to hepatocellular death. Strategies to improve the prognosis of I/R-induced liver injury via modulating autophagy are a current hot topic in experimental and clinical tests. Ischemic preconditioning (IPC) is divided into two steps, including cutting off hepatic inflow around the portal triad for 10–15 min and removing the clamp for another 10–15 min of reperfusion before liver transplantation [112]. Liu et al. recently reported that IPC and intestinal I/R prior to hepatic I/R confers protection against liver I/R-induced injury as demonstrated by the downregulation of serum aminotransferase and inflammatory cytokines via enhancing heme oxygenase-1 (HO-1)-mediated autophagy [113, 114]. Rapamycin-preconditioned MSCs enhanced the migration into the injured site and anti-inflammatory properties via regulation of the CXCR4/CXCL12 axis, thus affording additional protection against liver I/R injury [115]. In contrast, overexpression of HO-1 obviously increased the levels of autophagy-related proteins, ERK and p-ERK, but decreased the levels of mTOR and p-mTOR to increase autophagy in MSCs; then, HO-1-MSCs protected against I/R-induced injury in liver grafts following reduced-size liver transplantation [116]. Administering heat shock protein (HSP)-overexpressing MSCs significantly decreased the levels of serum aminotransferases and Suzuki scores and improved cell survival and histopathology in hepatic I/R rats by increasing autophagy and reducing apoptosis [117]. Activating autophagy prior to or after I/R improves the survival rate of MSCs in the liver and provides protective effects against liver I/R injury.

### I/R-induced retinal injury

Hypoxia or ischemia in the retina may induce sight-threatening disorders, including central retinal artery occlusion, diabetic retinopathy, and glaucoma, and result in the degeneration and loss of retinal ganglion cells. Retinal ischemia induced by elevated intraocular pressure upregulates the expression of LC3BII at the early phase of recovery, and posttreatment with rapamycin exerts no protection against the retinal lesion but increases the apoptosis of retinal cells [118]. However, Russo et al. demonstrated that rapamycin consistently upregulates autophagy and improves retinal ganglion cell survival in the hypoxic-ischemic retina, thus providing a potential therapy in retinas associated with ischemic stress [119]. Intravitreal transplantation of MSCs eliminated ischemic damage in the retina by suppressing apoptosis, attenuating the inflammatory response and vascular permeability, and preserving autophagy in a rat model [120]. Thus, MSC transplantation can rescue vision loss and serve as an effective treatment of ischemia-associated retinal degeneration.

### I/R-induced limb injury

Peripheral artery disease is characterized by pathophysiologic arterial narrowing and consequent stenosis, and accumulating evidence indicates that MSC transplantation can effectively restore blood flow in the ischemic limb. Volarevic et al. demonstrated that MSC transplantation could potentially serve as an alternative revascularization therapy for treating diabetic lower limb ischemia via pro-angiogenic effects [121]. Moreover, hypoxia-pretreated MSCs exhibited activated AMPK/mTOR signaling, enhanced autophagy, and improved pro-angiogenic effects, which improved the therapeutic effects in repairing lower limb function in ischemic diabetic rats [76]. Apelin significantly enhanced MSC survival in ischemic hindlimbs, restored hindlimb blood perfusion, and repaired limb functions by promoting autophagy, activating AMPK, and inhibiting mTOR in peripheral arterial disease [122]. In contrast, melatonin-pretreated MSCs enhanced neovascularization in a murine hindlimb ischemia model and promoted functional limb recovery via enhancing PrP^C^ expression and suppressing autophagy [59].

## Conclusions

In general, transplanted MSCs are confronted with a harsh environment in vivo, and the therapeutic application of MSCs is limited by their poor survival after engraftment. However, autophagy serves as a strict control to enhance the survival, engraftment, and paracrine effects of MSCs in vivo. Furthermore, this highly networked process offers protective effects against H/R- or I/R-induced injury via clearing away specific, no-longer-useful sets of macromolecules and providing additional energy. Targeting the potential mechanisms that modulate autophagy and thus control the anti-oxidative stress, anti-inflammation, and survival rate of MSCs will help improve the therapeutic effects of the engrafted MSCs in various I/R-induced injured tissues. Preconditioning MSCs by modulating autophagy via gene modification and specific activators or inhibitors may shed light on improving the regenerative capacity of MSCs in vitro and in vivo.

## Abbreviations

AMI: Acute myocardial infarction
AMPK: AMP-activated protein kinase
Atg: Autophagy-related gene
ATV: Atorvastatin
CMA: Chaperone-mediated autophagy
CPT1C: Carnitine palmitoyltransferase 1 C
CXCR4: CXC chemokine receptor 4
DAMPs: Damage-associated molecular pattern molecules
ER: Endoplasmic reticulum
H/R: Hypoxia-reoxygenation
H/SD: Hypoxia and serum deprivation
H_2_O_2_: Hydrogen peroxide
HIF-1α: Hypoxia-inducible factor-1α
HO-1: Heme oxygenase-1
HSP: Heat shock protein
I/R: Ischemia-reperfusion
IPC: Ischemic preconditioning
MCAO: Middle cerebral artery occlusion
MIF: Migration inhibitory factor
MnSOD: Manganese superoxide dismutase
MSCs: Mesenchymal stem cells
mTOR: Mammalian target of rapamycin
mTORC1: mTOR complex 1
OGD: Oxygen-glucose deprivation
PEDF: Pigment epithelium-derived factor
PGE2: Prostaglandin E2
PI3K: Phosphatidylinositol-3-kinase
PrP^C^: Prion protein
RIP1: Receptor-interacting protein kinase 1
ROS: Reactive oxygen species
SDF-1: Stromal cell-derived factor-1
TBI: Traumatic brain injury
TGF: Transforming growth factor
TLRs: Toll-like receptors
ULK: UNC51-like kinase
